# *‘It’s far too complicated’:* why fragmentation persists in global health

**DOI:** 10.1186/s12992-020-00592-1

**Published:** 2020-07-09

**Authors:** Neil Spicer, Irene Agyepong, Trygye Ottersen, Albrecht Jahn, Gorik Ooms

**Affiliations:** 1 London School of Hygiene & Tropical Medicine, 15-17 Tavistock Place, London, UK; 2 Ghana College of Physicians and Surgeons, PO Box DD1, Dodowa Health Research Centre, Dodowa, Accra, Ghana; 3 Norwegian Institute of Public Health, PO Box 222 Skoyen, 0213 Oslo, Norway; 4 Institute of Global Health, University of Heidelberg, Im Neuenheimer Feld 130.3, Heidelberg, Germany

**Keywords:** Synergies, Fragmentation, Coordination, Harmonisation, Alignment, Aid effectiveness, Interests, Accountability, Power

## Abstract

**Background:**

Despite many efforts to achieve better coordination, fragmentation is an enduring feature of the global health landscape that undermines the effectiveness of health programmes and threatens the attainment of the health-related Sustainable Development Goals. In this paper we identify and describe the multiple causes of fragmentation in development assistant for health at the global level. The study is of particular relevance since the emergence of new global health problems such as COVID-19 heightens the need for global health actors to work in coordinated ways. Our study is part of the *Lancet Commission on Synergies between Universal Health Coverage, Health Security and Health Promotion.*

**Methods:**

We used a mixed methods approach. This consisted of a non-systematic literature review of published papers in scientific journals, reports, books and websites. We also carried out twenty semi-structured expert interviews with individuals from bilateral and multilateral organisations, governments and academic and research institutions between April 2019 and December 2019.

**Results:**

We identified five distinct yet interconnected sets of factors causing fragmentation: proliferation of global health actors; problems of global leadership; divergent interests; problems of accountability; problems of power relations. We explain why global health actors struggle to harmonise their approaches and priorities, fail to align their work with low- and middle-income countries’ needs and why they continue to embrace funding instruments that create fragmentation.

**Conclusions:**

Many global actors are genuinely committed to addressing the problems of fragmentation, despite their complexity and interconnected nature. This paper aims to raise awareness and understanding of the causes of fragmentation and to help guide actors’ efforts in addressing the problems and moving to more synergistic approaches.

## Background

Five years have passed since the Sustainable Development Goals (SDGs) were launched in 2015. Yet, we are not on course to achieve the health-related SDGs and a lot more needs to be done to reach these targets. In the words of Dr. Tedros Adhannom Ghebreyesus, Director General of the World Health Organisation (WHO), fragmentation is an underlying problem: *‘ … the reality is, we’re off track to achieve these ambitious goals by 2030. Fragmentation, duplication and inefficiency are undermining progress’* [1]. The most recent global effort to address problems of fragmentation in global health is an initiative launched by a partnership of twelve multilateral organisations known as the *Global Action Plan: Stronger Collaboration, Better Health* [2]. The Plan was introduced at the highest level: it was inaugurated at the United Nations General Assembly in September 2019 and was publicly presented by all twelve partners at the World Health Summit in Berlin in October 2019. The Plan is depicted as a very important effort to focus attention on ways to accelerate and intensify actions to achieve the health-related SDGs. A critical principle is the need to embrace better coordination, that is to try to address problems of fragmentation, across the participating agencies and between the work of these agencies and low- and middle-income countries’ health priorities and strategies: *‘The overall objective of the Global Action Plan is to enhance collaboration among 12 global organizations engaged in health, development and humanitarian responses to accelerate country progress on the health-related SDG targets’* [2]. This is important as good coordination of development assistance for health (DAH) programmes is usually seen as contributing to health improvements. Good coordination helps to make more efficient use of scarce resources by avoiding duplication and gaps in health interventions and reduces the burden on low- and middle-income countries that occurs when multiple programmes, processes and systems are imposed on them by global health actors. It also ensures that global health actors’ activities better correspond with the priorities, strategies and systems of low- and middle-income countries receiving DAH [3, 4].

The Global Action Plan is by no means the first effort to mitigate fragmentation in DAH; indeed, there have been numerous, diverse efforts at the global level [5, 6]. In 1960 the Development Assistance Group, now known as the Development Assistance Committee, was formed with an aim of coordinating aid efforts among participating Organisation for Economic Cooperation and Development (OECD) member countries. Global health initiatives adopting a partnership approach, such as the Global Fund to Fight AIDS, Tuberculosis and Malaria (Global Fund) and many others, are sometimes presented as embodying strong coordination as they harness the collective finances, technical knowhow and creativity of multiple participating actors. Many such partnerships are, however, criticised for maintaining vertical health programmes and introducing parallel systems and processes [3, 4, 7, 8]. The Paris Declaration on Aid Effectiveness (2005) and the International Health Partnership Plus (IHP+) (2007) initiatives were widely supported by bilateral donors and other global health actors. They focussed global attention on the importance of improving aid effectiveness, and better coordination was presented as a critical principle, although implementation of these efforts is often seen as disappointing [6, 9, 10]. There are many other examples including Sector-Wide Approaches (SWAps) (1997), the ‘Three Ones’ principles (2004), ‘Health 8’ Agencies (2007), the Grand Bargain (2016) and UHC2030 (2016).

Despite these efforts to achieve better coordination, fragmentation remains an enduring feature of the global health landscape. It is acknowledged that substantial changes are needed in the ways global actors provide DAH; these ways of working have become highly entrenched over the decades since international development began after the Second World War. Fragmentation is one among many challenges that exist to achieving the health-related SDGs [7, 8, 11, 12].

Our study is part of the *Lancet Commission on Synergies between Universal Health Coverage, Health Security and Health Promotion* [13] that is aiming to identify ‘missed synergies’; that is, opportunities to address the problems of fragmentation and approaches to doing so. We define fragmentation as poor, or a lack of, coordination. In understanding coordination, we distinguish between the terms *harmonisation* and *alignment* embraced by the Paris Declaration on Aid Effectiveness. Harmonisation includes coordination of priorities, procedures and programmes and transparency among global health actors. Alignment means coordination between global health actors’ priorities, systems and interventions and those of low- and middle-income countries receiving DAH [14, 15]. In this paper we focus on global-level fragmentation, but not causes of fragmentation more directly linked to countries receiving DAH. We identify and describe the multiple causes of fragmentation at the global health level in setting agendas and formulating and implementing policies. We explain why global health actors struggle to harmonise their approaches and priorities, fail to align their work with the needs of low- and middle-income countries’ receiving DAH and why they continue to embrace funding instruments that create fragmentation.

While various aspects of fragmentation have been discussed elsewhere [for example 7,8,11,12], to the best of our knowledge, there is no other study or review that comprehensively brings together the breadth of factors causing fragmentation in global health. We believe all global health actors, including the architects of the Global Action Plan, need to intensify their efforts in tackling the issues presented in this paper if the SDGs are to be achieved. The aim of this paper is to help raise awareness and understanding of the multiple causes of fragmentation and guide actors in their pursuit of working in more coordinated ways. This paper is of current relevance, since the emergence of new and unexpected global health problems such as COVID-19 heightens the need for global health actors to embrace better coordination.

## Methods

We used a mixed methods approach consisting of a non-systematic literature review [16] and expert interviews [17]. Between April 2019 and December 2019, we carried out twenty semi-structured expert interviews with individuals from bilateral agencies, governments of low- and middle-income countries, academic institutions from high-income and low- and middle-income countries, research organisations and think tanks and multilateral agencies including global health initiatives. Respondents were women (*n* = 6) and men, and held senior managerial, technical and academic research posts. All had in-depth specialist knowledge of the issues of fragmentation at the global level, either programmatically or as publishing researchers, and represented low- and middle income countries (*n* = 7) and high-income countries. While our sample size of interviewees is relatively small, respondents had very high levels of expertise and highly relevant roles related to the topic we explored; hence we are confident we have effectively captured key issues relating to our aim. Interviews were conducted face-to-face, telephonically and using Skype and Zoom and were guided by a semi-structured topic guide. Adaptations were made to correspond with the specific expertise of some respondents. All interviews were conducted by NS and GO and were sound recorded and transcribed.

Our literature review was based on Google Scholar searches using multiple permutations of key terms: synergies; fragmentation; coordination; harmonisation; alignment; international development; health; aid effectiveness. Additionally, many of the sources we reviewed were identified from citations within the literature based on the web searches. Sources were selected on the basis of: a) relevance to the topic of fragmentation; and b) specifically relating to the health sector. In total we reviewed 68 articles and commentaries in academic journals, published reports and books and websites. Our literature review was limited to English language sources.

We adopted a health policy analysis approach: we assumed that policy actors and their power and interests, forms of governance, institutional rules and structures, ideas and values influence agenda setting, policy formulation and implementation [18]. We adopted an inductive approach in our systematic analysis of the interview transcripts and literature. Our thematic coding involved drawing out emerging themes from both the interview transcripts and literature, although the topic guide was initially informed by the literature and evolved over the course of the interviews.

We are presenting both the literature review and the interviews together since in combination they paint a richer and deeper picture of the multiple causes of fragmentation than if presented separately. Due to space limitations we have not been able to present all of the literature on the topic and all perspectives of our interviewees; we have been selective in our presentation of material while capturing the major points relevant to our aim.

Ethical approval was granted from the first author’s institution, the London School of Hygiene & Tropical Medicine, on 2nd April 2019.

## Results

**Fig. 1:**
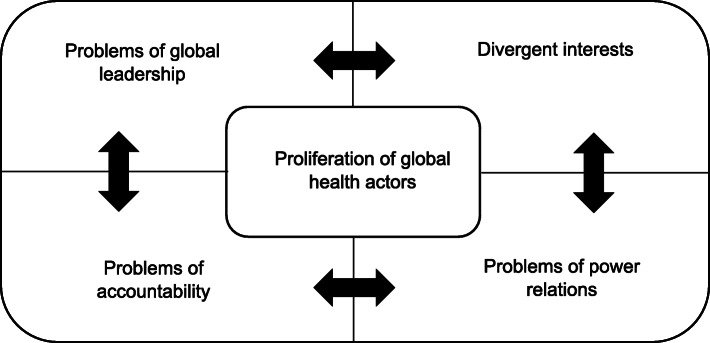
Factors causing fragmentation in global health

Based on themes presented in the literature and described by our interviewees, we drew out five distinct yet interconnected sets of factors causing fragmentation, represented in Fig. 1: proliferation of global health actors; problems of global leadership; divergent interests; problems of accountability; problems of power relations.

### Proliferation of global health actors: *‘*The Tower of Babel’

There has been a dramatic growth in the number and types of global health actors funding, managing and implementing health programmes since the creation of the United Nations (UN) system in 1945. This contributes to fragmentation as it makes it increasingly difficult to effectively coordinate global health efforts. Dodd et al. suggested that ‘...*there are now well over a hundred major international organizations involved in health, far more than in any other sector, and literally hundreds of channels for delivering health aid’* [19]. A total of 175 such actors were estimated by McColl in 2008 [20], while Hoffman and Cole [12] listed 203 global health actors in 2018, up from around fifty in 1960. A government interviewee reflected on the extent of fragmentation at the global level: *‘Well, fragmentation is everywhere! … the higher you go, the more you see fragmentation … ’,* while an academic interviewee commented: *‘ … we’re getting a lot of fragmentation and it’s getting worse as you get new entrants into the global health marketplace and there’s no overall plan or cohesion … ’.*

Global actors include the UN agencies, and specifically in the health sector, the WHO, the United Nations Children’s Fund (UNICEF) and the World Bank. The UN system of specialised agencies, funds and programmes has expanded over the years. UNAIDS was created in 1994 which replaced the WHO’s global programme on AIDS, the World Trade Organisation was established in 1995 and UN Women was launched in 2010. Bilateral agencies also have an important role in global health, and the numbers are growing. There are thirty high-income member countries of the Development Assistance Committee [21]. ‘South-south cooperation’ is becoming important; countries establishing bilateral agencies include Saudi Arabia, China, India and Brazil, and these actors are increasing in power and global influence [22, 23]. Fengler and Kharas listed at least 65 countries with official bilateral aid agencies or programmes [23]. A large proportion of countries receive aid from 25 donors or more, while some donors provide aid in more than fifty countries across health and other sectors [24, 25]. Additionally, large numbers of *inter*governmental organisations exist with a mandate for health such as the African Union, the Caribbean Community and Common Market and the European Union to name a few, together with growing numbers of research and knowledge generation organisations such as universities, consultancies and think tanks.

New types of actors are engaging in global health: *‘A dazzling kaleidoscopic environment’* according to Walt et al. [8]. Since the late 1990s and early 2000s, many global health partnerships and initiatives have emerged; the Global Fund, the Global Alliance for Vaccines and Immunisation (GAVI Alliance) and the United States President’s Emergency Plan for AIDS Relief (PEPFAR) are among the best known. A Lancet article published in 2009 listed no fewer than one hundred global health initiatives, almost all were vertical in that they focussed on specific health issues [26]. Commonly, high-income countries fund the Global Fund while at the same time maintaining their bilateral HIV/AIDS programmes [27]. Hence, despite the potential for bringing together existing actors to work on common aims, the launch of so many partnerships and initiatives has, ironically, added to the complexity.

Some commentators in the literature point to the vast numbers of civil society organisations involved in global health efforts. Estimates suggest there were 1983 in the early Twentieth Century, and by 2000 as many as 37,000 were estimated [28–30]. Fidler [31, 32] used the phases ‘unstructured plurality’ and ‘open source anarchy’ to highlight the ways governments are increasingly sharing their influence over global policy with the vast number of civil society organisations operating at global level, some of which are formally engaged in powerful decision making mechanisms such as the Global Fund’s Board. Many large transnational civil society organisations have substantial resources and influence in global health. Indeed, one type of civil society organisation has become particularly significant: private philanthropic foundations, and the Bill and Melinda Gates Foundation in particular: *‘Gates is probably the preeminent actor in shaping the global agenda’* an academic interviewee said. Private sector actors such as global pharmaceutical corporations are also becoming increasingly important in global health. Some engage in global health by becoming members of partnerships where they see commercial opportunities, to heighten their reputations and for altruistic reasons, including partnerships set up by the Gates Foundation [8, 33].

The growth in global health actors, is, in no small part, a response to globalising health problems, including communicable diseases rapidly crossing international boundaries [8]. It is often accepted that countries cannot work alone to address such problems [8], and hence it is important that global organisations and approaches exist. Disillusionment about the ability of existing global health actors to tackle these issues further underlines the need for additional actors bringing with them new resources, technologies and creativity [7].

The number of global health actors has grown to a point that the complexity intensifies the challenges of fragmentation. While the proliferation of actors and initiatives does not in of itself create fragmentation, coordination becomes more difficult with more actors. As one of our academic interviewees summed up: *‘I think just part of it is it’s far too complicated … ’.* Different global health actors, including multilateral and bilateral agencies, civil society organisations and philanthropic foundations and global health partnerships and initiatives commonly launch and fund their own programmes and interventions rather than funding existing ones. Each of these actors has their own interests, employs different financing instruments and follows different organisational rules and regulations, cultures, systems and processes such as reporting expectations and funding cycles. This incompatibility can make harmonisation more difficult in practice than it would be with fewer actors: multiple actors working collectively requires more effort than working individually and can slow progress in achieving health goals [24]. An academic interviewee observed: *‘You walk round the Palais du Nations and you think, how is anyone going to coordinate this … the Tower of Babel … that’s the challenge logistically … ’,* while an interviewee from a research organisation suggested: *‘ … it does take a lot of effort and time to meet other partners and understand their needs and agendas and trying to seek common ground … ’.* Additionally, global health actors’ priorities, rules and regulations, cultures, systems and processes are often imposed on and not aligned with those of low- and middle-income countries receiving their largesse. As there are so many global health actors, this can place a considerable burden on the health systems of those countries [7, 8, 11, 12, 34, 35]. Sridhar [34] explains:

*… lack of alignment of donors with the national approach, lack of harmonization among donors, and excessive transaction costs on recipient governments. Too often donors have their own ways of implementing initiatives in a country, thereby weakening national health strategies and systems …*

### Problems of global leadership: ‘The WHO stands on a crowded stage’

A second cause of fragmentation is the lack of effective leadership for global health. No single lead actor, institution or process exists that is able to harmonise the multitude of global health actors, and there is of course no ‘global government’ with jurisdiction over different countries in the same way that sovereign nations have national governments [7, 11, 36]. The actor with a lead role in global health is the UN’s specialised agency for health, the WHO, with its legal mandate for coordinating global health efforts and its normative role in leading on the setting of regulations and standards. Article 2 of the WHO’s constitution clarifies its coordination role: *‘In order to achieve its objective, the functions of the Organization shall be … to act as the directing and co-ordinating authority on international health work’* [37]. Despite member countries giving the WHO its mandate, opinions are divided about how strong the WHO is as the lead actor in global health. Some interviewees felt that the WHO continues to be held in high esteem in its normative role: *‘ … if they see something with a stamp of WHO they believe, they feel confident, they feel secure’* said a multilateral interviewee. Another interviewee suggested that the World Health Assembly continues to have some influence as the formal decision-making process for global health: *‘WHA is in theory the supreme legislative body because it does have treaty writing powers [with] the framework convention as the classic example … ’.*

The WHO’s difficulties in fulfilling its coordination role are, however, frequently commented on. Firstly, there are many other global actors with power and resources influencing global health, making coordination increasingly challenging for any single organisation [8]. Frenk and Moon [11] observe: *‘ … the WHO stands on a crowded stage; though once seen as the sole authority on global health, the WHO is now surrounded by diverse actors’.* Secondly, while the WHO’s formal coordination role remains, other actors’ ideas and approaches have challenged the WHO’s power and leadership. For example, UNICEF’s selective primary healthcare programme was a counter to the WHO’s ideas of ‘comprehensive’ primary healthcare. The World Bank has been famously called the *‘8000 lb gorilla’* in global health [38], a reference to its substantial resources, expertise, power and influence [8]. The World Bank’s ascendancy in the field of health is often linked to the influential 1993 World Development Report ‘Investing in Health’, which challenged the ideas of primary healthcare and universal health coverage advocated by the WHO, and displaced them with ideas of efficiency and the role of the market in the health sector: *‘ … we would have said in the past … the primacy was WHO. And [then] the Bank came along after 1993, the World Development Report … ’* captured an academic interviewee. More recently, the 2013–2016 Ebola epidemic in West Africa was seen as further undermining the WHO’s reputation as an effective leader [39]. The Gates Foundation has become an important funder of the WHO, the World Bank and indeed many other global health actors. One academic interviewee argued that the foundation is becoming a new lead actor in global health: *‘Without a doubt the global agenda is … extremely heavily influenced and shaped by the foundation … because of the money, but also because of the strategic approach which the Gates Foundation uses its money to leverage other actors’.*

Thirdly, the WHO and other UN agencies are sometimes criticised for having internal organisational problems that have reduced their power. An academic interviewee captured this point: ‘*The old institutions like WHO and the World Bank - many people have said they are not fit for purpose, they have been inefficient, governed poorly … ’.* Indeed, the failings of existing global health actors have prompted the creation of new actors and partnerships in global health, which in turn have challenged the WHO’s leadership [40]. An academic interviewee made this point when talking about the creation of the Global Fund and UNAIDS: *‘These [actors] were all in part [created] because of the perception that WHO as an institution couldn’t do it. We could have given money to WHO for Aids, TB and malaria but we didn’t’*. Fourthly, the WHO’s limited effectiveness as a global health leader is undermined by inadequate resources to meet its mandate in the context of new global health challenges such as HIV/AIDS, infectious disease pandemics and non-communicable diseases, coupled with pressure from its donors, each with different agendas and expectations, and with substantial control over its budget and priorities [40, 41].

Fifthly, and more broadly, some high-income countries see their national sovereignty, and hence their power and ability to further their own interests, as being threatened by strong health global actors and institutions such as the UN agencies and other multilateral efforts such as the Global Fund and similar partnerships and initiatives. Hence, they tend to be cautious in their support, or indeed have an interest in maintaining the fragmented global order. An academic interviewee explained: *‘They are usually trying to undermine [the global health architecture] to be perfectly honest, because they are afraid of conceding sovereignty. I mean they want to assert influence, but it is better the whole thing is weak rather than strong’.* This explains some high-income countries’ lack of willingness to loosen control of their funds on which the WHO depends. A government interviewee commented: *‘ … the World Health Organisation is a donor driven agency … 80% of its budget comes from donations … the best thing is for the WHO to be more focussed … don’t be dragged by donors!’.* An interviewee from a multilateral organisation added: *‘ … WHO ends up with its hands tied – not able to do much because of the conditions put to them … ’.* This does, of course, vary between high-income countries; some European countries are described as being fairly supportive of a strong global architecture compared to the United States: *‘ … the US isn’t ready to invest in one global fund for everything because they’ll have the feeling they’ll lose control … Europeans maybe a bit more, but not all of them and with certain conditions’* an interviewee from a bilateral donor explained.

### Divergent interests: ‘It’s tied to trade and security and influence’

A third cause of fragmentation is global health actors’ self-interests, and therefore their tendency to adopt priorities and approaches that do not always align with those of low- and middle-income countries receiving DAH. Indeed, the interests of global health actors can be divergent, competing and therefore incompatible with one another, making it difficult to harmonise the different priorities and approaches they adopt. Interests reflect very different histories, political climates, norms, values and cultures among countries contributing DAH [11, 42–44]. Health agendas have reflected and indeed contributed to the playing out of wider geopolitical agendas; an academic interviewee gave an example: *‘Alma Ata was the Soviet Union saying, hey you Americans, you can put a man on the moon, but you can’t provide healthcare for all you people … ’.* More contemporary rivalry exists between the United States, the European Union and China: *‘ … you have a bit of a global pissing match going on in terms of the multi-polar governance system between the US, the European Union system, China … ’.*

The interests of some global health actors appear to be predominantly altruistic. Such interests tend to be linked to DAH that is justified from a human rights perspective and when the aim is to improve health equity in low- and middle-income countries receiving DAH, and hence embraces ideas of justice, ethics and morality [44]. Similarly, charitable interests are served when rich countries feel they need to provide ‘relief’ for populations of low- and middle-income countries or to assuage a feeling of guilt for past colonial exploitation [43, 45, 46]. However, many of our interviewees and commentators in the literature admit that DAH is primarily, although not entirely, driven by the interests of high-income countries [24, 45]. A government interviewee summarised this perspective: *‘ … self-interest is the most challenging issue in the integration of different fragments … every agency has its own interests … only when their interests are in the same direction...harmonisation can occur. If not, they move in their own direction and they have conflict everywhere’.* High-income countries’ vested interests are often served through maintaining more fragmented approaches to DAH including bilateral funding and earmarking funds where the donor country has more control than when supporting multilateral approaches, despite evidence that the latter can be more beneficial for recipient countries in promoting economic growth and health impacts [47–50]. Gulrajani [50] summarises: *‘Bilateral donor interests appear to skew the aid allocation process in favour of strategic and political considerations, as opposed to country need or potential for development impact’.* An interviewee from a bilateral donor agency clarified: *‘We now have to show we are in the interests of [our country] in what we do – it’s a request from our ministers … even the word solidarity seems to be outdated in certain political circles’.*

Interviewees suggested that the health agendas of some bilateral donors have responded to the broad political shift to the right in the United States and Europe where nationalism, fear of immigration and lack of altruism are increasingly visible: *‘It’s getting touchy to sell development aid nowadays with our respective governments. I mean the political wind is blowing in another direction’* explained an interviewee from a bilateral donor agency. This means some high-income countries have explicitly justified DAH – to their own populations at least – on self-interest grounds. For example, responding to criticism about wasteful spending on international development, the UK’s Department for International Development openly admits that its aid budget benefits the UK: *‘Our aid commitment - which is enshrined in law - increases Britain’s global influence and allows us to shape the world around us. This is a win for the developing world and win for the UK too’* [51]. Our interviewees also observed that nationalistic concerns about immigration have made the idea of global health security more politically attractive since people crossing sovereign borders are assumed to transmit communicable diseases.

Global health security is often characterised as serving the interests of high-income countries by protecting their own citizens and economies. Hence, funding is usually directed towards communicable diseases in low- and middle-countries that might spread to high-income countries, rather than necessarily aligning with the priorities of countries receiving DAH [43, 44]. As an interviewee from a research organisation clarified: *‘I think that’s one of the features of Global Health Security isn’t it? It’s driven by, to some extent, by your interest to protect your own nation, your own interests … to kind of protect yourself from threats that might transmit across borders … ’.* The United States is often criticised for espousing this interest in many of its health programmes: *‘It comes down to political stances on certain issues … It would be difficult, for example, for the US to be as interested in UHC as they are in global health security … ’,* one interviewee from a research organisation explained. Health programmes can also serve foreign policy interests by bolstering the international reputation of high-income countries and strengthening their diplomatic relations with, and political influence over, countries receiving aid rather than necessarily aligning with the priorities of those countries [43, 46]. An academic interviewee captured this idea: *‘Hillary Clinton when she was Secretary of State, she coined the term “smart diplomacy”. Which is basically you do good to be liked and to get more, sort of, influence’.* It might also be argued that even health efforts that appear to serve altruistic interests can further foreign policy interests. Talking about PEPFAR an academic interviewee said: *‘I think George Bush genuinely believed it was a moral calling. But it had enormous positive benefits for the United States in terms of diplomacy and friendship and even security – particularly in sub-Saharan Africa’.*

The economic interests of high-income countries can also be served when health programmes promote trade and business investment with low- and middle-income countries receiving DAH, including expanding and creating new markets for products and services created in high-income countries, and access to natural resources [43, 44]. Indeed, it is assumed that countries receiving DAH will be beholden to high-income countries, and hence more likely to agree to such arrangements [45]. Very high levels of HIV/AIDS funding have been linked with underlying economic interests; one of our multilateral interviewees summarised: ‘*It was affecting the economies and it was everywhere … people who were dying were everywhere, and it was affecting … the big companies … because the workers were dying because of HIV/AIDS. And the economic consequences which were becoming serious … ’.* It is also argued that structural adjustment policies, with their aim of reconfiguring countries’ health sectors into more market orientated ones, helped to facilitate the entry of companies from high-income countries, including pharmaceutical corporations, private healthcare providers and insurers, into the markets of low- and middle-income countries [52]. And of course, the negative impacts on health and the damage caused to low- and middle-income country health systems by structural adjustment policies have been acknowledged, including weakening governments’ roles in planning, coordination and regulation – and thereby contributing to fragmentation [52].

Most high-income countries, to some degree, embrace economic interests, although the extent this is emphasised or acknowledged varies. Some countries continue to provide primarily ‘tied aid’ explicitly where it is expected that a proportion of aid used is spent on products produced in the donor country. Talking about China’s broader international development strategy, an academic interviewee explained: *‘I think Belt and Road is more nakedly mercantile – it’s intent is to increase trade routes from China … it’s tried to trade and security and influence … ’.* Other countries have tried to limit this; the UK’s 2002 International Development Act, for example, made tied aid illegal.

Finally, there is the phenomenon of ‘phantom aid’. A substantial proportion of DAH takes the form of professional staff and consultants’ salaries, administration and transaction costs, meetings and conferences. There are vast numbers of people employed by the aid industry and so, organisations have a strong interest in maintaining their existence, thereby contributing the proliferation described earlier [45, 53]. Indeed, Rogerson et al. [54] noted that after fifty years of aid no major global health actor, including UN agencies, had closed or merged. An academic interviewee commented: *‘I think fragmentation is good for many actors. If you just think about GAVI and the Global Fund, these are big institutions that employ large numbers of people and sustain careers … so, there’s always vested interests in the system you create … ’*.

### Problems of accountability: ‘I see a certain reluctance to more transparency’

A fourth cause of fragmentation stems from global health actors’ unbalanced accountability, that is weak accountability to governments and populations in low- and middle-income countries receiving their DAH contributions, while being more accountable to the high-income governments and taxpayers that fund them. Bilateral agencies are required to report to their country governments, and multilateral agencies, which are accountable to member states, tend to follow the edicts of high-income countries providing most funding. Health programme implementers, often civil society organisations, are primarily accountable to their donors [11]. Global health actors and programme implementers are under pressure to deliver results, that is attribute health impacts to their efforts; *‘flag raising’* as one multilateral interviewee put it. Hence, bilateral programmes and short-term, vertical programmes and projects can be attractive as they are more amenable to measuring rapid effects that are attributable to specific inputs than multilateral approaches or health systems strengthening efforts with longer term, unclear outcomes [6]. An interviewee from a bilateral agency commented: *‘I think most of the donors are under pressure to show what they do with tax payers money – some more than others … ’.* An interviewee from a think tank explained: *‘People are looking for short-term wins … they want to get a five-year win rather than a thirty-year win’.*

A consequence of these issues is that harmonisation can be undesirable as individual actors commonly fund their own parallel health programmes based on bilateral and vertical approaches to enable them to attribute impacts to their own, rather than collective, efforts: *‘ … this has intensified over the last decade … the sense of needing to deliver results … often that … becomes apparent in a vertical … programme’* an interviewee from a research organisation observed. Global health actors are increasingly scrutinised on the health impacts of their spending. An interviewee representing a European bilateral donor agency said that considerable accountability for spending was expected in their country and that aid was more thoroughly debated in parliament and by the press than previously. An academic interviewee talking about the US remarked: *‘ … accountability has a very important role … they want to be able to count how many lives saved … ’*. The same interviewee said about the UK: *‘ … lots of quantitative targets … that’s much easier if you take on AIDS or sleeping sickness or malnutrition … you can follow the money much more easily, you can count, have accountability … ’.*

Closely linked to this, is the tendency of global health actors to focus on vertical programmes addressing specific diseases or health issues since they are easier to ‘sell’ in high-income countries funding them than broader health systems activities because politicians, the media and populations can readily understand and relate to them. An academic interviewee summarised this point: *‘It’s easier to get money for something very concrete compared to something that’s building the [health] system’.* ‘Glamourous’ health issues tend to gain particular attention [7], with obvious examples being HIV/AIDS, malaria, tuberculosis and maternal, newborn and child health, and there it is clear skewing global funding towards these issues. For example, based on 2017 estimates, HIV/AIDS received 24% of DAH, despite being ranked eighth leading cause of early death globally, while health systems strengthening received 11%, down from 16% in 2001 [55, 56]. Effective advocacy has helped to leverage huge levels of funding for vertical HIV/AIDS programmes [7], while more recently polio eradication that is heavily promoted by the Gates Foundation continues to eclipse efforts to promote health systems strengthening: *‘There are very powerful lobbies lobbying to give funds to other areas’,* an interviewee from a think tank noted.

Because global health actors are in competition, they often lack transparency about their activities with each other and with the governments of low- and middle-income countries receiving DAH, again making coordination undesirable. An interviewee from a bilateral donor agency suggested: *‘ … I see a certain reluctance to more transparency … it can apply to recipient countries, but it could also apply to so called donors … ’.* Information also tends to flow from monitoring and research conducted in low- and middle-income countries receiving DAH to high-income countries funding DAH, including reports and academic papers commissioned by global health actors that cannot always be accessed in low- and middle-income countries. This makes it more difficult for those countries to effectively coordinate multiple, non-transparent global health actors within their overall health plans and strategies, and to hold them to account for their activities [7]. While these issues apply to bilateral and multilateral agencies, non-state actors, including philanthropic foundations and other civil society organisations as well as businesses, experts and journalists tend can have especially unclear roles, obligations and hence lines of accountability. According to Reich [57], foundations in particular have: *‘ … too much power to set public agendas, without sufficient public oversight and input*’.

There have been multiple efforts to reduce fragmentation in global health. While it is common for global health actors to enter into global commitments to reduce fragmentation such as the Paris Declaration on Aid Effectiveness and the International Health Partnership *Plus,* this is done so on a voluntary rather than binding basis, which is a weak mechanism for holding them accountable for failures or shortcomings. An interviewee from a research organisation said: *‘ … they are basically pieces of paper that don’t really have much traction or meaning … ’.* Hence, to be effective these global efforts would need to have stronger accountability mechanisms built in: *‘ … how do you give this initiative some teeth, how do you build accountability into this so that people feel that they need to do something?’* the same interviewee queried. The fact that there are very many agreements, declarations and commitments [5, 6], has, ironically, added to the proliferation of the global health architecture described earlier. Many efforts are relatively ephemeral; some are launched, and new ones launched soon after, making it difficult for those efforts to mature and yield results [6]. Without a protagonist, efforts do not survive: *‘I think IHP was very much linked to Gordon Brown … so when he was out it disappeared’* said an academic interviewee. An additional problem is that limited resources have been committed to implement global efforts: *‘The Paris Declaration … did play an important role … but it was not followed by a continuous provision of resources for the countries to implement their own programmes … that is the major deficiency’*, according to the same interviewee. It is therefore not surprising that most of these global agreements, declarations and commitments have not achieved or fully achieved their aims.

Individuals as well as organisations often seek to attribute results to their efforts. Hence, a cause of fragmentation can be the clash of ideas, values and interests of leaders of global organisations, including philanthropists with high levels of power to decide what their organisations do [29, 43, 58]. An academic interviewee argued: *‘It’s not policy, it’s personalities … you know, people have to demonstrate they’ve done something – the endless discussions about logos and dominance … people bolstering their own positions, unfortunately … ’.*

### Problems of power relations: ‘Dependent on playing by donors’ rules’

A fifth cause of fragmentation relates to the power relations that exist between rich and poor countries. Some critics argue that DAH is part of an apparatus that helps to maintain unequal power relations by holding back or even damaging the economies of low- and middle-income countries while serving the interests of high-income countries and their multinational corporations [59–62]. For example, the donation of money and commodities can undermine businesses in low- and middle-income countries and creates a relationship of dependency, fails to build strong national institutions and systems and serves to support corrupt and non-democratically elected leaders [45, 59–62]. An academic interviewee captured this problem: ‘ … *if you don’t have strong institutions that are able to track, manage, the flow of money into a country, you just create more opportunities for money to leak out of the system, for certain people to benefit … ’.* In turn, assumptions about corruption within countries receiving DAH, whether well-founded or not, justify global health actors’ lack of faith in the ability of low- and middle-income governments to effectively implement their own health programmes. This can validate ‘top-down’ approaches that reinforce fragmentation, such as channelling funding through civil society organisations, avoiding pooled funding and budget support approaches and introducing parallel systems and processes.

Some writers adopting a ‘dependency theory’ approach argue that aid can make recipient countries dependent on high-income countries, and therefore more likely to follow their political ideologies and agendas, although this is a contentious issue. This was most obvious during the Cold War, and it has been noted that countries supporting the West’s war on terror have enjoyed more generous aid receipts [45]. The result is, some global health actors emphasise their own priorities rather than aligning with those of low- and middle-income countries. A multilateral interviewee said: ‘ *… they play with money … to persuade or make other countries or other partners believe that the way they are suggesting is the best way to go. If there’s something that doesn’t go according to what they thought, then the pressure through money is used’.* Dependency causes fragmentation as low- and middle-income countries receiving DAH tend to be in a weak negotiating position for fear of losing essential largesse, and hence are more likely to follow global health actors’ priorities and interests than insisting they align with their own priorities, programmes and systems [24, 63].

The extent to which global health actors determine the health priorities of low- and middle-income countries varies. The lowest income and most fragile states with weak health systems, low capacities, weak institutions, and often high levels of corruption, tend to have limited latitude to manage the priorities and programmes of multiple, competing global health actors and their implementers. An interviewee from a research organisation explained: *‘ … more fragile and lower-income countries, essentially the less domestic revenue generation they have, the more dependent they are on donor funding and therefore more dependent on playing by donors’ rules’.* Middle-income countries tend to have more influence on global health actors than low-income countries do. A multilateral interviewee gave an example: *‘South Africa is quite good. Because South Africa is less and less dependent from external resources’.* Some low-income countries have, according to our interviewees, been successful at coordinating multiple global health actors’ health programmes, despite receiving high levels of DAH, including Ethiopia and Rwanda. Interviewees agreed that critical factors were strong leadership, long-term country health plans and strong coordination mechanisms. A multilateral interviewee commenting on Ethiopia said: *‘If you get the leadership and consistency in policy application then you can minimise most of the damage caused by the global actors’.*

Other writers argue that DAH can reproduce power relations as it discursively reproduces ideas around the ‘helplessness’ of poor countries and their people who are assumed to depend on Western charity. Indeed, the terms ‘developing country’ and ‘third world’ suggest that it is inevitable that low- and middle-income countries will aspire to become ‘developed’ by following high-income countries’ development models, which usually means embracing neoliberal principles [64]. Research conducted by academics, consultants and experts, whether intentionally or not, can, potentially, reproduce the dominant discourses of powerful global health actors, or at the very least fail to criticise and hold them to account [65]. An interviewee from a multilateral organisation summarised: *‘ … a growing group of academia … pushing many papers for the sake of trying to make others believe what they think should be happening … there’s no check for that … trying to play into others’ agendas and not genuinely trying to address the problem …’.* Nevertheless, many global health actors now claim to have distanced themselves from these ideas and language has evolved that seems to suggest this is happening, with terms such as ‘development partners’, ‘low- and middle-income countries’ and ‘global south’ having become widely accepted parlance.

## Discussion

Our analysis suggests that tackling fragmentation is a daunting challenge given the multiple, interconnected causes. However, we believe there is a genuine intention among many global health actors to acknowledge and address these problems. Indeed, fragmentation was publicly presented by the WHO’s current Director General as a critical barrier to achieving the health-related SDGs, and there have been many high-profile efforts to reduce fragmentation, and now, improving coordination is a central aim of the Global Action Plan.

Fragmentation remains a sticky problem, and the COVID-19 crisis makes it more important than ever to tackle. Can anything be done to address or mitigate the problems we outline in this paper? Proliferation of global health actors is a major factor. The global health landscape is becoming more and more complex with the addition of new global health actors, many of which fund their own separate programmes and interventions rather than contributing to existing ones or working collectively. Ironically, the growth in efforts to reduce fragmentation has further complicated the global health landscape [5, 6, 8]. We suggest global health actors now need to avoid adding to this complexity. In most cases, they should aim to contribute to existing programmes and interventions, build on existing declarations, targets and initiatives, and strengthen existing actors, institutions and processes rather than launching new and potentially ephemeral initiatives. As much as possible, high-income countries should channel funding through multilateral actors and initiatives, and thereby aim to reduce the number of parallel bilateral programmes they fund.

Another cause of fragmentation relates to problems of global health leadership created by multiple challenges, including some high-income countries seeing strong global leadership, institutions and multilateralism as a threat to their national sovereignty and global influence. This is an ongoing issue; for example, in early 2020 the United States accused the WHO leadership of bias towards China amid the COVID-19 crisis and threatened to withhold its donations. The twelve multilateral signatories of the Global Action Plan have signalled their strong resolve to improve coordination. However, the Plan may be less impactful than it could be without the very substantial resources, power and influence of the actors that are not signatories to the Plan, not least the major funders of global health. Multilateral actors need to challenge the sovereignty problem by pushing the major health funders, at least those that are willing, to embrace the principles of the Plan and work closely with them to achieve its goals.

It is perhaps inevitable that high-income countries will, to some extent, continue to serve their own interests when engaging in DAH activities. Fragmentation stems from global health actors’ divergent interests, and the lack of alignment between their interests and the priorities and systems of countries receiving DAH [45, 47, 50]. Some commentators are arguing that different global health actors can pragmatically work together to achieve common shared objectives, despite espousing divergent interests [44]. Yet the problem of divergent interests appears to be intensifying; current trends towards the political right in Europe and North America threaten the generosity of development assistance and willingness to embrace collective approaches to tacking health problems. It is becoming more publicly acceptable to present DAH as serving the interests of high-income countries. The COVID-19 pandemic may heighten the importance that some high-income countries attach to global health security in order to protect their own populations. China’s influence over global health agendas is likely to increase as it extends its role as a global health funder. Current trends suggest economic interests will be a major motivation for China’s contributions to DAH, and it is unclear whether the country will embrace synergised ways of working in global health. The problem of self-interests means that it is common for many of the negative externalities of fragmentation to be passed on to low- and middle-income countries receiving DAH. Efforts are needed to readdress the balance of benefits and costs: low- and middle-income countries need to receive more benefits and bear fewer costs of DAH. Ultimately this means global health actors need to redouble their commitments to ensuring their work is well-aligned with low- and middle-income countries’ national priorities. Rather than launching their own parallel programmes, DAH should be channelled through low- and middle-income countries’ strategies, systems and programmes, for example, by embracing government-led coordination mechanisms and pooled funding mechanisms. Low- and middle-income countries could also be supported to increase their capacities to critically assess the impacts and effects of global health actors’ funding. Having better information should enable them to more effectively manage, and potentially challenge, multiple DAH programmes, ultimately leading to better health outcomes.

Unbalanced accountability is another cause of fragmentation. Strong accountability of global health actors to high-income countries undermines harmonisation, while weak accountability to low- and middle-income countries receiving DAH undermines alignment [11]. Moreover, the many global efforts at improving coordination have tended to not be successful for multiple reasons including weak accountability mechanisms, the large number and ephemerality of initiatives, and limited resources for their implementation [6]. Strengthening accountability mechanisms could help to reduce fragmentation. These might include government-led mutual accountability mechanisms such as common monitoring frameworks promoted by the International Health Partnership *Plus* that started to yield results in some low- and middle-income countries involved in that initiative [10, 66]. The global monitoring partnership known as IHP + *Results* helped identify successes and reveal limited progress, and hence put pressure on participating actors and countries to follow through on their commitments [6, 9, 10, 66].

Finally, power relations create fragmentation as many low- and middle-income countries receiving DAH find it difficult to insist on global health actors aligning their activities with their priorities and systems [24, 63]. Yet, some low-income countries receiving high levels of development assistance, such as Ethiopia and Rwanda, have been able to ensure DAH meets their needs, as well as manage the activities of multiple global health actors in their countries – or at least to mitigate some of the damage. Ethiopia benefits from strong leadership and country ownership in the health sector, the existence of strong, long-term government-led national health strategies and having in place strong donor coordination mechanisms [67].

The causes of fragmentation described in this paper are interconnected and this makes them difficult to address. Proliferation does not in of itself create fragmentation, but it does mean that effective coordination is more difficult than ever before. It also makes it more challenging for the WHO to fulfil its leadership role. The large number of global health actors, coupled with divergent self-interests and unbalanced accountability, results in poor harmonisation and alignment that burdens low- and middle-income countries with weak health systems. High-income countries’ self-interests also undermine global leadership and reproduce power relations with low- and middle-income countries. Ultimately, major changes in behaviour are needed. As one of our multilateral interviews argued: *‘ … there needs to be a paradigm shift! It’s not going to happen overnight because people are used to working in certain ways … ’.* If there is no paradigm shift at the global level it will be up to the low- and middle-income countries receiving DAH to find ways to better manage the activities of global health actors in their countries, or at least to better mitigate some of the damage.

### Limitations

One limitation of our study is the relatively small sample size of respondents. The number of interviewees is, however, consistent with what is expected from in-depth qualitative studies involving expert informants and we argue that the calibre of our interviewees and the depth of their knowledge makes a strong basis for the results we present. We also believe we reached a good level of saturation as our interviews progressed. Nevertheless, we are not claiming that our findings are definitive; inevitably our own perspectives and experiences have shaped how we conducted the study and interpreted the data. Despite our interviewees’ expertise, we may not have captured all factors relating to the causes of fragmentation. Indeed, we only draw on English language sources and hence our analysis may exclude issues reported in the non-English literature. A second limitation is that we have presented broad-brush patterns. Substantial variations exist in the interests and approaches of different global health actors and the global health landscape is constantly changing. Certain issues are controversial; it is likely some readers will disagree with some of our arguments. However, we maintain we have presented the major perspectives that appear in the literature and were held by our expert interviewees, even if some of our findings and conclusions do not apply to all global health actors. Thirdly, in this paper we focus on issues at the ‘global level’. The *Lancet Commission on Synergies between Universal Health Coverage, Health Security and Health Promotion* is also exploring the nature, causes and effects of fragmentation within high-income countries and within low- and middle-income countries receiving DAH. Finally, our underlying assumption in this paper is that fragmentation has negative consequences, especially within low- and middle-income countries. However, some of our interviewees and commentators in the literature suggest that fragmentation is not necessarily negative and can even be positive; for example, by facilitating competition among global health actors and their implementers leading to more innovative solutions [68].

## Conclusion

Fragmentation undermines the effectiveness of health programmes supported by global health actors and threatens the attainment of the health-related Sustainable Development Goals. This paper describes five distinct yet interconnected sets of factors causing fragmentation: proliferation of global health actors; problems of global leadership; divergent interests; problems of accountability; problems of power relations. Many global health actors are genuinely committed to reducing fragmentation, despite the complexity of the problem and its causes. This paper aims to raise awareness and understanding of the causes and to help guide actors’ efforts in addressing the problems and moving to more synergistic approaches. New global health problems such as COVID-19 heightens the need for global health actors to better coordinate their efforts in working towards common goals.

## Data Availability

In order to be consistent with our ethical approval and agreements about confidentiality and anonymity with our interviewees it is not appropriate to make the qualitative dataset supporting the conclusions of this article publicly available.
